# Correction: Effects of Type of Health Insurance Coverage on Colorectal Cancer Survival in Puerto Rico: A Population-Based Study

**DOI:** 10.1371/journal.pone.0104581

**Published:** 2014-07-30

**Authors:** The PLOS ONE Staff

There is an error in the Financial Disclosure (FD) statement for this article. Please refer to the correct FD statement below:

This work is supported by federal funds from the National Program of Cancer Registries (NPCR Award Number 5U58-DP 003863-02; URL: http://www.cdc.gov/cancer/npcr/) to the Puerto Rico Central Cancer Registry (PRCCR), by the University of Puerto Rico Comprehensive Cancer Center and by the Department of Health Services Administration, Graduate School of Public Health, Medical Sciences Campus, University of Puerto Rico. The ideas and opinions expressed herein are those of the authors, and endorsement by the funders is not intended nor should it be inferred. The funders had no role in the study design, data collection and analysis, decision to publish, or preparation of the manuscript.
